# A bioinspired and chemically defined alternative to dimethyl sulfoxide for the cryopreservation of human hematopoietic stem cells

**DOI:** 10.1038/s41409-021-01368-w

**Published:** 2021-06-21

**Authors:** Renata Gilfanova, Andrea Callegari, Adam Childs, Gaomai Yang, Miranda Luarca, Alan G. Gutierrez, Karla I. Medina, Justin Mai, Alvin Hui, Mark Kline, Xiaoxi Wei, Philip J. Norris, Marcus O. Muench

**Affiliations:** 1 Vitalant Research Institute, San Francisco, CA USA; 2 X-Therma Inc., Richmond, CA USA; 3 Department of Laboratory Medicine, University of California, San Francisco, CA USA

**Keywords:** Stem-cell research, Translational research

## Abstract

The cryopreservation of hematopoietic cells using dimethyl sulfoxide (DMSO) and serum is a common procedure used in transplantation. However, DMSO has clinical and biological side effects due to its toxicity, and serum introduces variation and safety risks. Inspired by natural antifreeze proteins, a novel class of ice-interactive cryoprotectants was developed. The corresponding DMSO-, protein-, and serum-free cryopreservation media candidates were screened through a series of biological assays using human cell lines, peripheral blood cells, and bone marrow cells. XT-Thrive-A and XT-Thrive-B were identified as lead candidates to rival cryopreservation with 10% DMSO in serum based on post-thaw cell survival and short-term proliferation assays. The effectiveness of the novel cryopreservation media in freezing hematopoietic stem cells from human whole bone marrow was assessed by extreme limiting dilution analysis in immunodeficient mice. Stem cell frequencies were measured 12 weeks after transplant based on bone marrow engraftment of erythroid, myeloid, B-lymphoid, and CD34^+^ progenitors measured by flow cytometry. The recovered numbers of cryopreserved stem cells were similar among XT-Thrive A, XT-Thrive B, and DMSO with serum groups. These findings show that cryoprotectants developed through biomimicry of natural antifreeze proteins offers a substitute for DMSO-based media for the cryopreservation of hematopoietic stem cells.

## Introduction

Hematopoietic stem cell (HSC) transplantation is often a last resort for patients battling various inherited or acquired conditions [1, 2]. With donors coming from international and intercontinental sources, safe and effective cell storage is an essential step in the transplant procedure to accommodate graft inventory, logistics, transportation, quality control testing, and major histocompatibility matching of the donors and recipients [3–5].

Living cells experience injury when exposed to freezing temperatures [6]. Water molecules organize into ice crystals and impair cells by mechanically damaging organelles and membranes as well as creating an electrolyte imbalance outside of the cells, causing an outflux of water and dehydration. To prevent cell death cryoprotective agents (CPAs) are used that interfere with water crystallization, with 10% dimethyl sulfoxide (DMSO) in a solution with a high content of serum being standard for cryopreservation [7, 8].

Although an effective CPA, DMSO is also toxic to cells and to patients receiving cell products containing DMSO [9, 10]. Common clinical side effects of DMSO include nausea, vomiting, and hypotension [11], but other toxicities such as central nervous system [12–14], cardiovascular [15], respiratory [16, 17], renal [3], hemolytic [18], hepatotoxic, and even fatalities have been reported [19]. Nearly 100% of bone marrow transplant recipients receiving DMSO-cryopreserved cells suffer side effects or serious complications during infusion [20]. As a chemically active small molecule, DMSO interacts with many cellular and signaling pathways and affects the epigenetic profile of cells even at concentrations as low as 0.1% [21]. Removal of DMSO before transplantation can lead to cell loss [22] and is time-consuming, cumbersome, and does not fall in line with minimal manipulation guidelines set by the FDA. Moreover, as a strong solvent, DMSO leaches and etches plastic transfusion tubing as well as other containers, which increases risk for cGMP processes [23].

Efforts have been made to find alternatives to DMSO with limited success [24–27]. The common practice of addition of animal and human sera also introduces variables in a research setting in addition to batch-to-batch variation, potential biohazards, supply chain issues, ethical, and geographic restrictions that come with its use [28, 29]. Various other proteins are also used by manufacturers of freezing media to optimize performance, but the instability of protein components leads to reduced shelf-life [30].

With demand for safe and effective alternatives to DMSO, researchers have looked to the natural world to study adaptation and survival in subfreezing temperatures [27, 31]. Herein, novel cryoprotectants inspired by natural antifreeze protein structure were tested on hematopoietic cells. The fully synthetic cryoprotectants control ice formation and are non-toxic, chemically stable, and are protein-free. Candidate formulations were compared to 10% DMSO in serum for use during liquid nitrogen storage (−196 °C) [32].

## Materials and methods

### Ethics Statement

Adult peripheral blood mononuclear cells (PBMCs) were recovered from Trima leukoreduction chambers discarded during the normal course of volunteer blood donations [33]. As these cells were not obtained for the purpose of experimentation and the donors are anonymous, use of these cells was not considered human subject research requiring Institutional Review Board approval and subject consent.

Midgestation bone marrow (BM) was harvested from long bones as described [34]. Samples were obtained with written informed consent and the approval of the Institutional Review Board at the University of California San Francisco (IRB#10-00768) and in accordance with the Declaration of Helsinki. Specimens were donated anonymously at San Francisco General Hospital.

Mice were transplanted with approval and oversight of the Institutional Animal Care and Use Committee at Covance Laboratories Inc. (San Carlos, CA; Animal Welfare Assurance A3367-01) under protocol IAC 2235/ANS 2509. Investigators adhered to the Animal Welfare Act Regulations and other Federal statues relating to the animals, and experiments involving animals, and the principles set forth in the current version of the Guide for Care and Use of Laboratory Animals, National Research Council. Mice were maintained under specific-pathogen free conditions as previously detailed [35] and euthanized according to the recommendations of the American Veterinary Medical Association. The standard diet was exchanged for irradiated Global 2018 rodent diet with 4100 ppm Uniprim (Envigo) ≥3 days before irradiation and the mice were maintained on this diet for a month.

### Isolation of PBMCs

PBMCs were harvested from Trima leukoreduction chambers [33]. Recovered cells were diluted 1:4 with phosphate buffered saline (PBS) and density separation was performed using Lymphoprep according to manufacturer’s instructions (Alere Technologies AS, Norway). Light-density PBMCs were washed with PBS twice and suspended in Iscove’s modified Dulbecco’s medium (IMDM) with 10% of fetal bovine serum (FBS; Gibco, Thermo Fisher Scientific, Grand Island, NY).

### Cell counting and flow cytometric analysis

Cells were counted on a Nexcelom Bioscience Cellometer K2 (Lawrence, MA). Viability was measured based on acridine orange/propidium iodide viability staining. Flow cytometry was performed [36] using antibodies listed in Supplementary Table 1.

### Cryopreservation and cell recovery

Cryopreservation was performed using novel chemically-defined DMSO-, serum-, and protein-free biomimetic cryopreservation formulas produced by X-Therma Inc. (Richmond, CA), which were tested against 10% DMSO in heat inactivated FBS (90%). Novel biomimetic CPAs contained additional nutrients, such as saccharides for energy, salts to maintain ion balance, membrane stabilizers to strengthen the membrane at cold temperatures, antioxidants to scavenge free radicals, and other molecules to maintain proper osmotic balance [37]. These X-Therma formulations were used as provided. A new ampule of DMSO (Product #D2650, Sigma Aldrich, St. Louis, MO) was used for each experiment. Typically, cells were aliquoted at 10 × 10^6^ / vial, centrifuged, depleted of media and suspended in 1 ml of cryoprotectant. In the following 5 min, the cryovials were placed into CoolCell FTS30 freezing containers (Corning, Corning, NY) and transferred to a −80 °C freezer. According to the manufacturer, these containers provided a −1 °C / minute freezing rate. After 24 h the cells were moved to liquid nitrogen for 8–22 days.

Cells were thawed quickly in a 37 °C bath until only a small piece of ice was left in the vial, with subsequent mixing of the cells with warm PBS. PBMCs and BM cells were washed by centrifugation at 4 °C 200 × *g* for 5 min to remove DMSO, as well as novel CPAs for consistency, to reduce the effects of DMSO in culture or transplanted mice.

### In vitro PBMC proliferation assay

PBMCs were cryopreserved at 1.5 × 10^6^ cells per vial. After 6–14 days in liquid nitrogen, PBMCs were thawed and cell recovery and viability assessed immediately after thawing as well as after 24 h of culture in IMDM with 10% FBS. Data for different PBMC types were acquired using flow cytometry. Cell recovery was calculated as percent of cells after thawing relative to input. In addition, the number of cells harvested after culture was compared to cultured cells from both not cryopreserved (fresh cells) and cryopreserved cells. Thus, comparing the survival/proliferation potential of fresh and cryopreserved cells. For example, the number of B-cells harvested 1 day after culture of 1.5 × 10^6^ never-frozen PBMC’s was considered 100%, and the frequency of B-cells recovered from cultures of cryopreserved cells was calculated relative to this number.

Comparisons of the performance of novel cryopreservation media to DMSO controls were made using one-way ANOVA analysis using Dunnett’s multiple comparison test (Prism 8, GraphPad Software LLC). A stack graph combining the percent recoveries of all cell types was produced, providing simple visualization of the best performing cryopreservation media.

### TF-1 and Jurkat culture assay

TF-1 (ATCC® CRL-2003™) cells were cultivated in suspension in RPMI 1640, (Gibco) supplemented with 10% FBS, 5 mL Pen/Strep 100x, and 2 ng/ml recombinant human granulocyte-macrophage colony-stimulating factor (R&D Systems, Minneapolis, MN). Jurkat cells were maintained in the same medium without growth factor. For experiments, cells were suspended at 5 × 10^6^ cell/ml in 200 µl CPA in cryovials. CryoStor10 (CS10) (BioLife Solutions, Bothell, WA), a commercial cGMP grade CPA containing 10% DMSO with defined components, was used as a control. Vials were held on ice for 10 min, transferred into an alcohol-free foam freezing box, placed at −80 °C for 2 h, and then in liquid nitrogen storage. After 6 days, cryovials were rapidly thawed using a 37 °C water bath and the cells diluted with 800 µl of warm medium.

Viability of TF-1 was evaluated up to 2 days post-thaw using a cell viability and proliferation assay following the manufacturer protocol (Biotium, Fremont, CA). Briefly, a vial of 2.0 × 10^3^ cells was diluted 1:200, plated in triplicate in 96-well plates, and incubated with Calcein AM, EthD, and NucBlue for 30 min at 37 °C. Fluorescent live (green) and dead cells (red) were counted on a fluorescence microscope (EVOS m5000, Thermo Fisher Scientific) using an automated counting program.

The alamarBlue assay (Thermo Fisher, Invitrogen, Waltham, MA) was used to evaluate metabolically active Jurkat cells after cryopreservation. Following the manufacturer protocol, 10 × 10^4^ cells from the diluted vial were plated in triplicate in 96-well plates together with a standard curve. The alamarBlue was added to a final concentration of 10% and incubated for 2 h at 37 °C. Fluorescence was measured at an excitation wavelength of 530–560 nm and an emission wavelength at 590 nm by plate reader (Infinite F200, Tecan, Männedorf, Switzerland). Cell numbers were extrapolated using the standard curve. One-Way ANOVA statistical analysis was performed using Minitab software.

### In vitro BM proliferation assay

Cryopreserved whole BM was quickly thawed in a warm water bath, live cell numbers and viability frequencies determined, and then cultured in 24-well plates at 1 × 10^6^ – 1.5 × 10^6^ cells / 1 ml using serum-free medium [38] supported by recombinant-human interleukin 3 (20 ng/ml) and kit ligand (50 ng/ml). After 72 h, cells were counted and analyzed by flow cytometry. X-Therma cryopreservation media candidates were compared to 10% DMSO in 90% serum using one-way ANOVA with Dunnett’s multiple comparison test.

### Stem cell engraftment of mice

Founder NOD.Cg-*Prkdc*^*scid*^ *Il2rg*^*tm1Wjl*^ Tg(PGK1-KITLG*220)441Daw/SzJ mice were obtained from the Jackson Laboratory (Bar Harbor, ME) and bred at Vitalant Research Institute. Adults of both sexes were sublethally irradiated with 175 cGy and, 3 h later, transplanted with 1 × 10^6^ cells, 3 × 10^5^ cells, 1 × 10^5^ cells or 3 × 10^4^ cells as described [36].

On day 84 post-transplant, engraftment in the BM and spleen was analyzed by flow cytometry as published [36]. Human β2-microglobulin (B2M) and mouse TER-119, CD45, H-2K^d^ markers were used to aid separation of cells by species. An engrafted mouse was defined as being positive for 3 hematopoietic lineages: myeloid—CD33^+^, erythroid—CD235^+^, B-lymphoid—CD19^+^ as well as CD34^+^ hematopoietic precursors. Alternatively, engraftment was defined by the presence of CD34^++^CD133^+^ cells. An untransplanted mouse was analyzed in the same way and used as a control to define lack of staining for human cells. A mouse with more than 5 positive events for a human antigen was considered to be “positive” for that marker [39]. ELDA was used to quantify HSC frequencies [40].

## Results

### Testing of biomimetic CPA formulas on PBMC

**Fig. 1:**
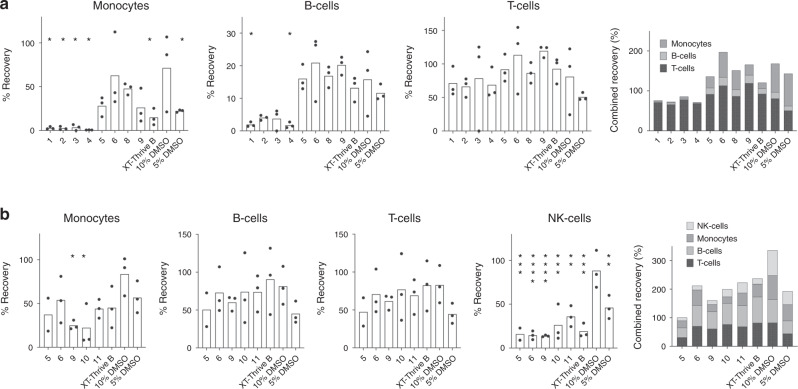
Recovery of PBMC cryopreserved in X-Therma formulations or 10% DMSO. **a** Cell recoveries of cryopreserved monocytes, B-cells, and T-cells after 24 h culture. 100% represents the number of cells harvested after 24 h cultures of never frozen (fresh) cells. **b** Results from a second experiment using additional formulations with NK-cells also analyzed. Data are shown as mean for *n* = 3 cultures for each formulation. Stacked histograms (right most graphs) represent the combined recovery-percentages of each cell type to visualize overall cell recovery for each CPA. Asterisks above the graph represent statistically significant differences compared to 10% DMSO: *P < 0.05, ** *P* < 0.01, ***P < 0.001, and ****P < 0.0001.

Biomimetic DMSO-free CPA formulas were tested using PBMC, a source of various blood cell lineages that may respond differently to cryopreservation. Testing was done blinded with CPA candidates coded by X-Therma before being tested at Vitalant Research Institute. PBMC were stained for monocytes (CD3^-^CD56^-^CD14^+^), B-cells (CD19^+^), and T-cells (CD3^+^) 24 h after culture. PBMCs cryopreserved in ten candidate CPAs showed differential post-thaw recoveries, with some showing comparable cryoprotective efficiency to 10% DMSO in serum (Fig. 1a). Monocyte recovery was similar to 10% DMSO in serum when frozen in CPA candidates 5, 6, 8, and 9, but significantly diminished in the other groups. There was no significant difference from 10% DMSO for lymphocyte recoveries except for significant B cell loses using formulations 1 and 4.

Promising candidates were re-tested along with further optimized candidates (Fig. 1b). NK-cells (CD19^−^CD3^−^CD14^−^CD56^+^) were also examined but fared best when cryopreserved in 10% DMSO. For B-cells, T-cells, and sometimes monocytes, the new CPA candidates performed similarly to serum-based 10% DMSO. Overall, the XT-Thrive B formulation was the best at preserving leukocytes from among all the candidates tested in the second round of experiments.

### Formula selection using TF-1 cells

**Fig. 2:**
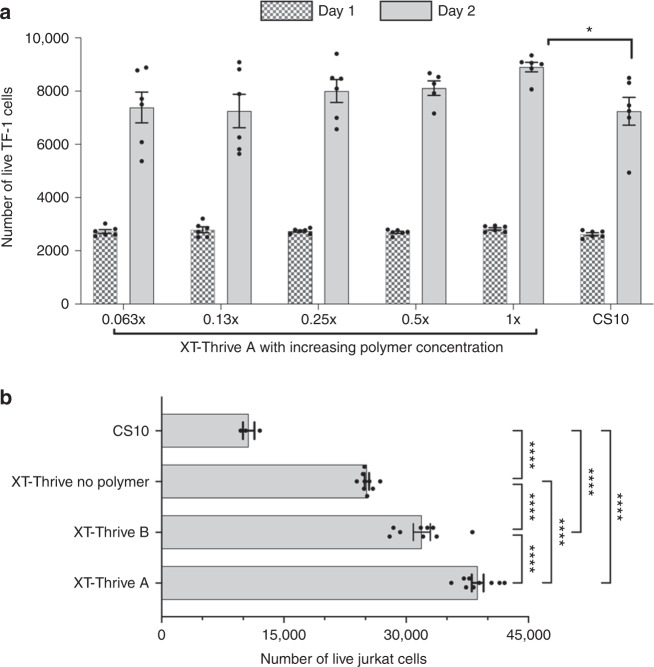
Cryopreservation of hematopoietic cell lines in XT-Thrive A and XT-Thrive B. **a** Proliferation of TF-1 cells measured as number of live cells after 1- and 2-days in culture. A commercial CPA containing 10% DMSO, CS10, was used as a control. Data are shown as mean ± SE (*n* = 6). **b** Proliferation of cryopreserved Jurkat cells during 2-days of culture. Data are shown as mean ± SE (*n* = 4). *P < 0.05, ****P < 0.0001.

Additional screening was performed using the human erythroleukemia cell line TF-1. These cells are CD34^+^ and were chosen as a model for hematopoietic precursors for screening of candidate CPAs. TF-1 cells preserved with XT-Thrive A grew more after 2 days in culture than cells frozen in CryoStor10 (Fig. 2a). Furthermore, a positive correlation was observed between the concentration of the biomimetic cryoprotectant used and proliferation (One-Way ANOVA; *P* = 0.033). The 1X concentration was selected for subsequent experiments with BM cells.

### Evaluation in Jurkat cells

Based on the above results, XT-Thrive A and XT-Thrive B were compared further using Jurkat cells, a human T-cell line. As shown in Fig. 2b, cryostorage in XT-Thrive A and B formulations improved cell viability 2 days after culture compared to CryoStor10 (One-Way ANOVA; *P* < 0.0001). A control without polymer was also examined. As expected, the performance of this formulation was inferior to XT-Thrive A and B, but still performed better than CryoStor10, indicating that XT-Thrive components in the cryomedia contribute to the cryopreservation of the cells in the absence of added polymer.

### Increased in vitro proliferation of hematopoietic precursors when cryopreserved with novel cryomatrix formulations

**Fig. 3:**
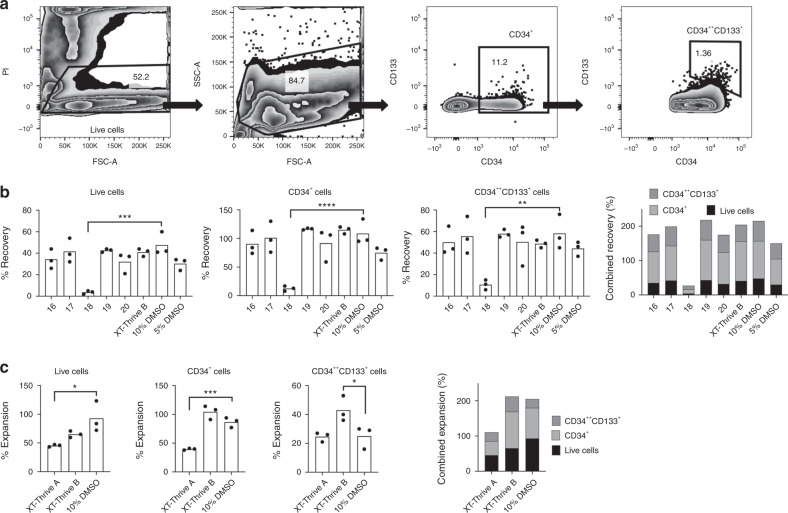
Cryopreserved BM recovery and proliferation after culture. **a** The gating strategy used to define total live, total CD34^+^, and CD34^++^CD133^+^ BM cells is shown for a sample cryopreserved in XT-Thrive B analyzed after 3 days in culture. **b**, **c** Percent cell recoveries for all live cells and the total CD34^+^ and CD34^++^CD133^+^ subpopulations are shown for two experiments. 100% for each population represents the number of never frozen cells plated at the same cell density as thawed cells and enumerated after 3 days. Stacked histograms (right) represent overall CPA performance. Data are shown as mean of *n* = 3 cultures for each formulation. *P < 0.05, ** *P* < 0.01, ***P < 0.001, and ****P < 0.0001. PI propidium iodine, FSC-A Forward Scatter Area, SSC-A Side Scatter Area.

The recovery and growth of hematopoietic progenitors from cryopreserved BM was compared for a number of CPA candidates and 10% DMSO in serum. Total cell recoveries were evaluated as well as the numbers of CD34^+^ hematopoietic precursors and the subpopulation of CD34^++^CD133^+^ cells enriched for HSC (Fig. 3a). BM cryopreserved in XT-Thrive B yielded cell recoveries that did not statistically differ from 10% DMSO (Fig. 3b). In a second experiment, the numbers of CD34^++^CD133^+^ cells from the XT-Thrive B group were higher than the DMSO controls (*P* = 0.03) (Fig. 3c). BM cells frozen in XT-Thrive A had recovery of CD34^++^CD133^+^ cells comparable to the DMSO group, although the total number of CD34^+^ cells was lower (*P* < 0.001).

### HSC cryopreservation performance measured by ELDA

**Fig. 4:**
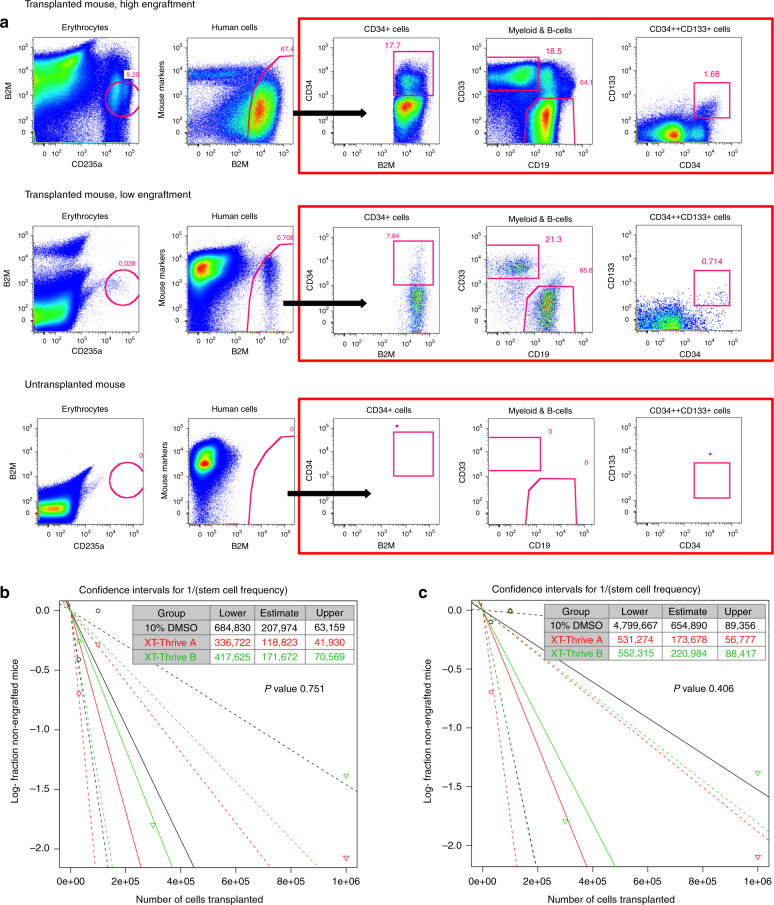
Limiting dilution analysis of transplanted cryopreserved HSC. **a** Flow cytometric analysis of mice engrafted with human BM 12 weeks after transplantation. An untransplanted mouse was used as a control. Mice positive for human CD34, CD33, CD19, and CD235a antigens or CD34^++^CD133^+^ staining, using the gates shown, were considered to be engrafted. Mice were transplanted with different doses of BM cryopreserved in XT-Thrive A, XT-Thrive B, or 10% DMSO in serum. **b** ELDA indicates similar HSC frequencies and overlapping confidence intervals as indicated by the insignificant *P* value for a test of overall differences in HSC frequencies, based on the 4-marker analysis, between any of the groups. **c** Similar results of ELDA were found based on CD34^++^CD133^+^ engraftment alone.

ELDA was used to compare the abilities of XT-Thrive A, XT-Thrive B, and 10% DMSO in serum to cryostore HSCs capable of long-term (12 weeks) hematopoietic reconstitution in immunodeficient mice (Fig. 4a). There was no significant difference in the estimated HSC frequencies based on the engraftment of erythroid, B-lymphoid, myeloid, and progenitor engraftment among the three groups (Fig. 4b). The highest frequency of HSC was obtained by cryopreservation with XT-Thrive A, followed by the XT-Thrive B and 10% DMSO. The frequencies of human CD34^+^ cells in mouse BM as well as the overall frequency of human cells engrafted were also analyzed (Supplementary Data Fig S1) and confirmed the similarities in engraftment potential between the XT-Thrive A and the controls using DMSO.

HSC engraftment was also analyzed based on the presence of CD34^++^CD133^+^ primitive progenitors (Fig. 4a). ELDA resulted in lowered estimates of HSC frequencies overall (Fig. 4c). No significant differences in the efficacies of the different cryopreservation agents were observed.

## Discussion

An alternative to DMSO for the cryopreservation of HSCs that can preserve stem cell function with improved safety for patients is highly desirable in order to meet the demands of bioprocessing and manufacturing for cell and genetic therapies. DMSO has been used to reduce the formation of ice in cells stored in liquid nitrogen since 1959 [41]. Along with effectively preserving cells, it also, unfortunately, can impair the functional recovery of cells [21] and is responsible for a variety of clinical side effects when administered to patients [20]. We investigated a novel class of chemically-defined antifreeze protein mimetics for HSC storage that were free of DMSO, serum, and other proteins. Candidate formulations were screened and selected by comparing their effectiveness on the cryopreservation of PBMCs and TF-1 cells. In-vitro tests of cryopreserved human BM showed similar performance of XT-Thrive A and XT-Thrive B when compared to 10% DMSO in 90% serum. Most importantly, the numbers of BM engrafting HSCs were maintained by XT-Thrive A and XT-Thrive B, demonstrating the efficacy of the new cryomedia for freezing HSCs.

A period of post-thaw cell culture is essential for apoptosis to progress [42]. Immediate post-thaw viability assays, such as Trypan Blue staining, often yield inaccurate measures of true cryoinjury to cells [43]. We assessed both immediate post-thaw viability of PBMCs as well as total live cell counts after 24 h culture to allow for completion of programmed cell death. Expectedly, overall cell viability was generally promising when measured immediately, but cultured cryopreserved cells compared to fresh never-frozen samples often showed lower recoveries of B-cells, monocytes, and NK-cells. Interestingly, all X-Therma formulations showed comparable performance for T-cell recoveries. The use of these novel CPAs in CAR-T cell therapy warrants investigation considering current cryopreservation and manufacturing challenges for these cells [44, 45]. The screening of CPAs using PBMCs, the TF-1 cell line, and short-term BM cultures led to the selection of two leading candidates with superior performance, XT-Thrive A and XT-Thrive B, for further investigation on transplantable HSCs.

HSCs are defined by their capacity to provide long-term, multilineage hematopoiesis in-vivo. In addition, we defined HSC engraftment by the presence of CD34 antigen [46]. In this work mice were considered engrafted by HSC when erythroid, myeloid, B-lymphoid, and progenitor engraftment were all observed. In addition, we defined HSC engraftment by the presence of CD34^++^CD133^+^ cells. ELDA was used to estimate HSC frequencies using either of the measurements of engraftment [40], which indicated that XT-Thrive A and XT-Thrive B formulations preserved HSCs at levels comparable to DMSO and serum using either of the measurements of engraftment. Effective cryopreservation of hematopoietic progenitors and HSC is critical for ensuring rapid and lasting engraftment after transplantation. Higher CD34^+^ cell doses reduce clinical complications and shorten engraftment times [47, 48], whereas low doses can lead to graft failure or life-threatening complications [49]. The numbers of HSCs transplanted are also believed to affect the overall life-long durability of the hematopoietic graft [50, 51].

Short-term proliferation assays on thawed cells showed lower cell yields compared to fresh cells no matter which CPAs was used. This decrease in hematopoietic activity could likely impact patient engraftment using cryopreserved cells. However, access to fresh cells for transplantation is supply limited or unavailable for some HSC sources pointing out the need to further improve cryopreservation methods [3]. Overall, we demonstrated that X-Therma formulations using biomimetic CPAs were minimally equivalent to DMSO in serum for HSC cryopreservation without introducing procedural complexity and may offer a clinically safer alternative to DMSO-based solutions.

## Supplementary information

Supplmental Materials
