# Supplementary material for: A bioinspired and chemically defined alternative to dimethyl sulfoxide for the cryopreservation of human hematopoietic stem cells
Source: Bone Marrow Transplant. 2021 Jun 21;56(11):2644–50. doi: 10.1038/s41409-021-01368-w (PMC8563414; doi:10.1038/s41409-021-01368-w)
Supplement: Supplementary file 1 — Supplmental Materials [file 41409_2021_1368_MOESM1_ESM.pdf]

Supplementary Table 1. List of antibodies used in this study.

Antibody	Fluorophore	Company	Catalog No.	Clone	Isotype
Antibodies recognizing human antigens					
CD3	FITC	BioLegend	300306	HIT3	IgG2a
CD14	PE	BioLegend	325606	HCD14	IgG1
CD19	PE-Cy7	BioLegend	302216	HIB19	IgG1
CD33	APC	BioLegend	303408	WM53	IgG1
CD34	PE	BioLegend	343506	581	IgG1
CD34	PE-Cy7	BioLegend	343516	581	IgG1
CD38	PE-Cy7	BioLegend	303516	HIT2	IgG1
CD38	PE	BioLegend	303506	HIT2	IgG1
CD41	FITC	BioLegend	303704	HIP8	IgG1
CD42b	APC	BioLegend	303912	HIP1	IgG1
CD56	APC	BioLegend	318310	HCD56	IgG1
CD133	APC	Miltenyi Biotec	130-090-826	AC133	IgG1
CD235a	PE	BioLegend	349106	HI264	IgG2a
β-2 microglobulin	FITC	BioLegend	316304	2M2	IgG1
HLA-DR	PE	BioLegend	307606	L243	IgG2a
Non-specific antibodies used as isotype controls					
IgG2a	FITV	BioLegend	400208	MOPC-173	
IgG1	APC	BioLegend	400120	MOPC-21	
IgG1	FITC	BioLegend	400108	MOPC-21	
IgG1	PE	BioLegend	400112	MOPC-21	
IgG1	PE-Cy7	BioLegend	400126	MOPC-21	
Antibodies recognizing mouse antigens					
CD45	Pacific Blue	BioLegend	103126	30-F11	IgG2b
TER119	Pacific Blue	BioLegend	116232	TER-119	IgG2b
H2KD	Pacific Blue	BioLegend	116616	SF1-1.1	IgG2b

Supplemental Data

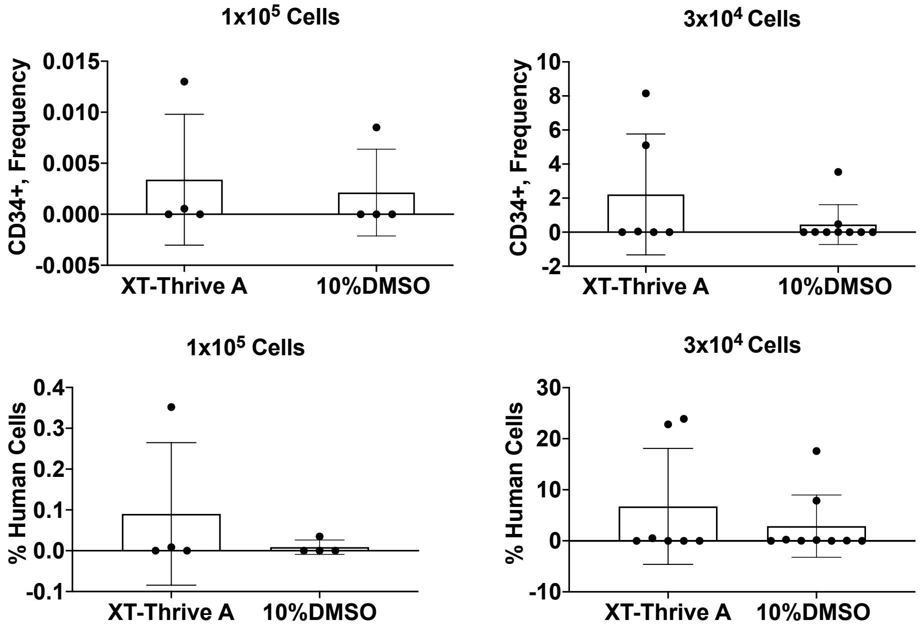

Supplementary Figure 1. **Frequency of human cells in NSG mice.** Frequencies of human $CD34^+$ cells and all human nucleated (β2-microglobulin$^+$) cells in mice transplanted with $1x10^5$ or $3x10^4$ human BM cells cryopreserved in XT-Thrive A or 10% DMSO. Data are from the limiting dilution experiment shown in Fig. 4. Each data point represents one engrafted mouse.
